# Landscape variables affecting the Himalayan red panda *Ailurus fulgens* occupancy in wet season along the mountains in Nepal

**DOI:** 10.1371/journal.pone.0243450

**Published:** 2020-12-11

**Authors:** Kanchan Thapa, Gokarna Jung Thapa, Damber Bista, Shant Raj Jnawali, Krishna Prasad Acharya, Kapil Khanal, Ram Chandra Kandel, Madhuri Karki Thapa, Saroj Shrestha, Sonam Tashi Lama, Netra Sharma Sapkota

**Affiliations:** 1 WWF Nepal, Baluwatar, Kathmandu, Nepal; 2 Red Panda Network, Baluwatar, Kathmandu, Nepal; 3 Ministry of Forests and Environment, Babarmahal, Kathmandu, Nepal; 4 Department of National Parks and Wildlife Conservation, Babarmahal, Kathmandu, Nepal; 5 Department of Forest and Soil Conservation, Kathmandu, Nepal; 6 United States Agency for International Development, Kathmandu, Nepal; Universidad Austral de Chile, CHILE

## Abstract

The Himalayan red panda is an endangered mammal endemic to Eastern Himalayan and South Western China. Data deficiency often hinders understanding of their spatial distribution and habitat use, which is critical for species conservation planning. We used sign surveys covering the entire potential red panda habitat over 22,453 km^2^ along the mid-hills and high mountains encompassing six conservation complexes in Nepal. To estimate red panda distribution using an occupancy framework, we walked 1,451 km along 446 sampled grid cells out of 4,631 grid cells in the wet season of 2016. We used single-species, single-season models to make inferences regarding covariates influencing detection and occupancy. We estimated the probability of detection and occupancy based on model-averaging techniques and drew predictive maps showing site-specific occupancy estimates. We observed red panda in 213 grid cells and found covariates such as elevation, distance to water sources, and bamboo cover influencing the occupancy. Red panda detection probability $\hat{p}\;(SE)$ estimated at 0.70 (0.02). We estimated red panda site occupancy (sampled grid cells) and landscape occupancy (across the potential habitat) $\hat{\Psi }\;(SE)$ at 0.48 (0.01) and 0.40 (0.02) respectively. The predictive map shows a site-specific variation in the spatial distribution of this arboreal species along the priority red panda conservation complexes. Data on their spatial distribution may serve as a baseline for future studies and are expected to aid in species conservation planning in priority conservation complexes.

## Introduction

Himalayan red panda (*Ailurus fulgens*) is a Himalayan mammal endemic to Eastern Himalayas and South Western China. Species range has declined by 50% in the past two decades and their conservation status has been reassessed from “threatened” to “endangered” by the IUCN Red List in 2015 [1]. Poaching, habitat loss, and degradation have been a major threat to species survival. This arboreal species is labelled as the next big black-market pet in the region. More than 121 red panda hides were confiscated in Nepal alone in the last decade (Central Investigation Bureau unpublished data) and the trend in the illegal trade of red panda pelts has increased significantly since 2008 [2]. Illegal wildlife trade is posing a pertinent threat which requires reliable data on the distribution of species to strategize protection measures during patrolling, management, and conservation actions [3].

Understanding species distribution and abundance are critical for setting appropriate management goals, monitoring effectiveness, informing policymakers, and other relevant stakeholders. Species distribution models are also valuable in addressing key biological questions, including the impacts of ecological and anthropogenic factors that influence distribution and habitat use for species of conservation concern [4]. Global red panda distribution is estimated to cover an area of 134,975 km^2^ [5] spread across five nations including Nepal. The Conservation Breeding Specialist Group (CBSG) conducted a red panda Population and Habitat Viability Analysis (PHVA) that identified six red panda conservation complexes in Nepal measuring an estimated area of 23,977 km^2^ [6]. Multiple studies have been focused on these complexes with conservation themes ranging from scale-dependent distribution, habitat use, and diet ecology etc. [7, 8]. Few empirical studies exist that quantify the distribution of the species. Thapa et. al. [5] focused on predicting red panda distribution along the entire range but based on presence-only data. In 2016, the Government of Nepal led a nationwide assessment as part of the flagship species monitoring program for enumerating red panda distribution. Only data that confirms their presence in nationwide assessment was limited to district wide distribution whereby red panda was recorded in 24 out of 37 potential districts. Acharya et al. [9] advocated for creating a special red panda conservation zone that ensures the conservation of a genetically viable population in the long run. The foremost step that helps in delineating the conservation zone has been quantifying the distribution of red panda. For this, habitat occupancy [10] has been proposed as robust and reliable metrics for estimation. Till now, there has been limited metrics available on the estimation of red panda occupancy along its range in Nepal. Nevertheless, rigorous occupancy modelling has been successfully tested in red panda at the landscape-level (Chitwan Annapurna Landscape) [11] and protected area level (Dhorpatan Hunting Reserve) [12] in the past. The spatial extent of the present study has been larger than the previous studies as they were confined in a relatively smaller area. This study has been scaled out to cover the entire range of red panda potential habitat identified along six conservation complexes based on large scale red panda sign survey.

Our main goal was to estimate and predict the spatial distribution of red panda built upon occupancy modelling framework [10]. Our objectives were: 1) to investigate the factors affecting the red panda occupancy probability using landscape-level covariates; and 2) to develop predictive red panda distribution maps based on occupancy estimates for the entire range in Nepal including six conservation complexes proposed by CBSG.

## Materials and methods

### Ethics statement

The study was conducted longitudinally along the red panda habitat in Nepal after gathering necessary research permits from the Department of National Parks and Wildlife Conservation (Ref no: 2072/073 Eco 237–2428) and Department of Forests and Soil Conservation (Ref no: 2072/073–1220). We used non-invasive method such as recording indirect signs left by animals, thus animal care and use committee approval was not required.

### Study area

**Fig 1:**
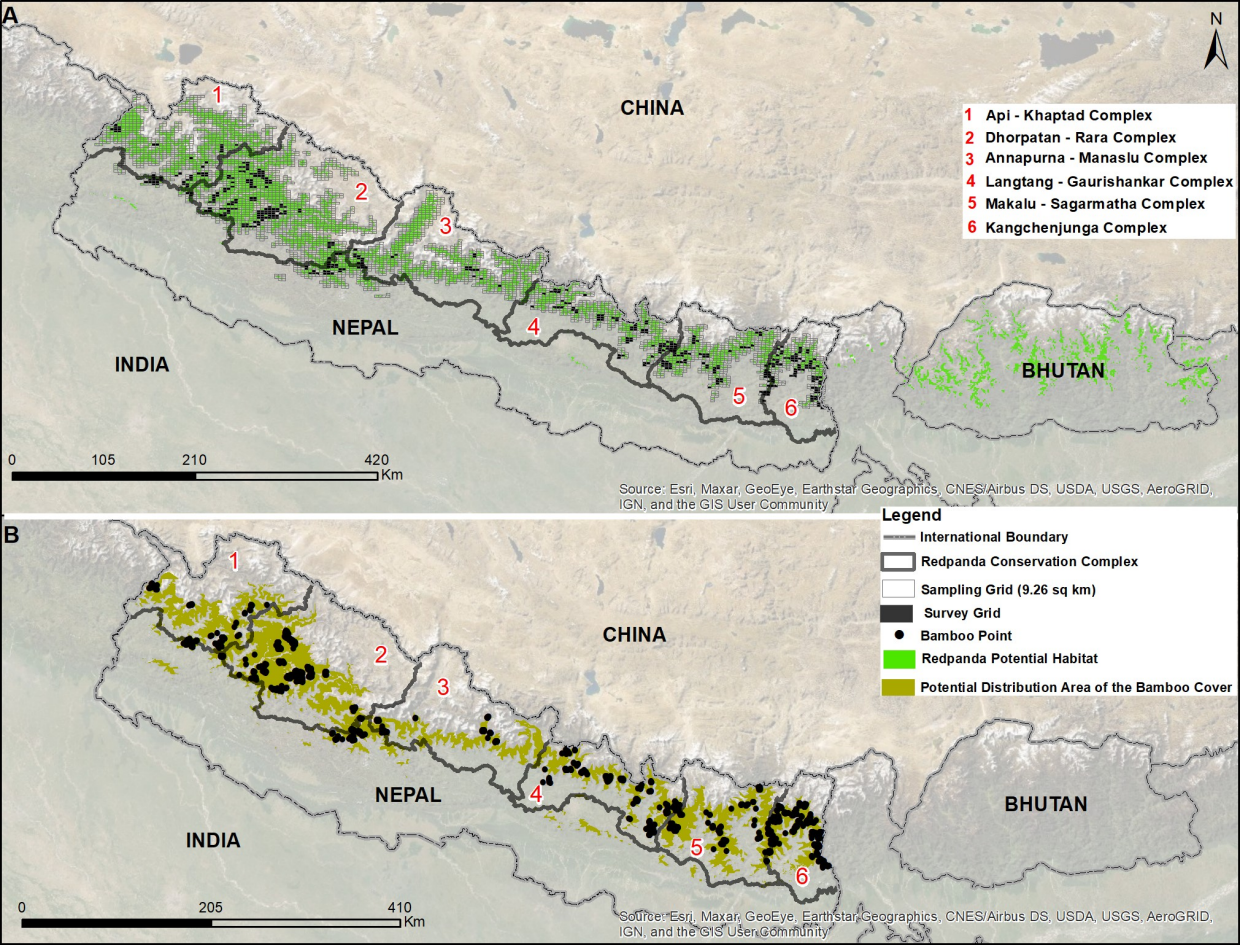
(A)-Study area showing matrices of major grid (each measuring 9.62 km^2^) spread across six conservation complexes in Nepal including red panda potential habitat along Nepal, India, and Bhutan. (B)-Distribution of the bamboo cover (BAM) from Maxent Modelling along with bamboo presence points (in black dots) along six conservation complexes in Nepal.

We conducted the study along the potential habitat of red panda in Nepal (N29.95 E80.67—N27.09 E88.00; Area: 22,453 km^2^, Fig 1). The potential habitat lies between 1,500 to 5,000 meter above sea level (masl) encompassing mid-hills (Area: 16,116 km^2^) and high mountains (Area: 6,429 km^2^) physiographic zones [11, 13]. Major red panda potential habitat includes montane forests (oak mixed, mixed broad‐leaf conifer, and conifer) with abundant bamboo thicket in the understory [14, 15] within the identified elevation range (2,000–4,000 masl) [6]. Generally, montane forests have Himalayan birch (*Betula utilis*) followed by east Himalayan fir (*Abies spectabilis*). Other forest patches include Himalayan larch forest (*Larix spp*.; deciduous subalpine habitat), the evergreen temperate coniferous forest (blue pine *Pinus wallichiana*) temperate broadleaf forest or lower temperate oak forest (*Quercus semecarpifolia*), and lower temperate conifer (*Picea-Tsuga*) or spruce forest (*Picea smithiana*) [16]. Total potential habitat in Nepal represents nearly 16.5% of the potential habitats available in North-East Asia (Nepal, India, Bhutan, Myanmar, and China) [7]. Potential habitat is embedded in six potential conservation complexes (from East to West) as Kangchenjunga Complex (total area: 7,173 km^2^; Protected Areas (PAs): Kanchenjunga Conservation Area; red panda habitat available: 694 km^2^), Makalu-Sagarmatha Complex (total area: 12,007 km^2^; PAs: Makalu Barun National Park, Sagarmatha National Park; red panda habitat available: 1,070 km^2^), Langtang-Gaurishankar Complex (total area: 10,853 km^2^; PAs: Gaurishankar Conservation Area, Langtang National Park; red panda habitat available: 973 km^2^); Annapurna-Manaslu Complex (total area: 15,592 km^2^; PAs: Annapurna Conservation Area, Manaslu Conservation Area; red panda habitat available: 1,333 km^2^), Dhorpatan-Rara Complex (total area: 20,490 km^2^; PAs: Dhorpatan Hunting Reserve and Rara National Park; red panda habitat available: 3,629 km^2^), and Api-Khaptad Complex (total area: 14,097 km^2^; PAs: Api Nampa Conservation Area, Khaptad National Park; red panda habitat available: 1,949 km^2^).

### Field design

This study is a by-product of the 2016 national assessment by the Government of Nepal to assess the occurrence of red pandas throughout the potential habitat identified in the PHVA workshop [6]. We employed occupancy modelling techniques using animal sign data collected to derive stronger inference by decomposing true absence from non-detection within a probabilistic framework [10]. The red panda has relatively small home ranges and maximum size estimated at 9.62 km^2^ recorded in the Langtang National Park located in Langtang Gaurishankar Complex [11, 17]. Additionally, this major grid cell size (~9.62 km^2^) circumscribes the expected daily movement based on home range size and movement rates reported at other field sites [7, 11, 12, 18]. We created a layer of major grid cells (~4361, each grid cell measuring 9.62 km^2^, Fig 1) in ArcGIS 10.2 and overlaid it with 22,453 km^2^ potential red panda habitat in Nepal [6] to investigate red panda occupancy and examine ecological and anthropogenic factors influencing it.

Sampled major grid cells (hereafter referred to as grid cell) were chosen based on the availability of more than 50% of potential red panda habitat within each grid cell. We sampled 446 grid cells (~10% of total grid cells), spread across the red panda habitat, sampling 6 spatial replicates and each replicate consisting 3–5 transects within each of a sub-grid measuring 1.6 km^2^. Division of the major grid cells into six sub-grid cells was primarily designed for the logistical reason (geographic complexity and terrain condition) and improving detection history of secretive animal. We chose for spatial replication over temporal [19] primarily for logistical reasons as done in previous studies for large mammals [20–23]. Adequate sign detections, replications and spatial coverage can be achieved with adequate survey efforts per grid cells and careful planning [20]. We fixed a total of 3–5 transects (defined here as search paths) ranging between 500 m to 1000 m, located along 100 m contours, were available within each sub-grid.

In each sub-grid cell, we searched for red panda signs: droppings (shiny greenish faeces when fresh), feeding signs (foraging marks on leaves usually 2 m above ground), scratch marks, fresh carcass, and footprints etc. Being arboreal (usually spends 86% of time resting on trees) [18], we also looked up on trees for possible detection through direct sighting. The survey was conducted once in pre-monsoon (June/July) and post-monsoon season (October) of 2016. Both the time (pre- and post-monsoon) were identified and defined here as the wet season. During the survey, detection and non-detection of animals were coded either a “1” for the presence or “0” for absence while walking on the fixed search path in each sub-grid cell. Due to maximum numbers of grid cells to be surveyed, low encounter rate of red panda signs, and ease in modelling process (convergence issue with lots of non-detection data “0” in detection history), we summarized detection and non-detection data from the fixed search path for each of the six sub-grid cell to develop the detection history. Thus, we have a maximum of 6 sampling occasions (1 to 6 spatial replicates) within each major grid cell. In the encounter histories matrix, in situations where there were incomplete survey histories a missing value entry (-) was incorporated into the matrix.

We divided the grid cells (study area) into three blocks to manage logistics. Selection of the first grid cell was random within each block. We randomly selected the starting point of the first transect within each grid cell (sub-grid cell) and followed a compass azimuth for each transect along the contours with a team comprising of field biologists accompanied by 2–3 skilled field personnel. Only fresh signs or direct sightings were collected to minimize false detections that could occur from the presence of a very old sign [24]. Age of a sign is difficult to ascertain, but our methods were conservative, and we did not include a sign that appeared old and weathered. The team searched for signs along and/or either side of the transects following probable routes (human/animal trails, fire lines, dirt roads) along the contour that was deemed to have a high likelihood of encountering the signs [25]. To optimize the detection, the team deviated from search routes on the ground (1–10 m) looking for signs and/or possible direct sighting along with the bamboos or trees branches but ensured uniformity in spatial coverage within each grid cell. The team completed surveying each grid cell on an average of 8 hours to assume spatial closure (i.e. to minimize the bias from the movement of animals from the surrounding grid cells) [26].

### Selection of covariates at the gid level

We aimed to estimate occupancy based on data collected from the sampled grid cell and extrapolated to the entire potential range of the species in the country. For country-level data analyses with occupancy models, there was a need of a covariate that explains a large proportion of variation in occupancy or abundance across space (e.g., environmental covariates), and that at least some sampling occurs along with the entire range of these covariates [27].

The landscape-level covariates were used to investigate its influence on detection and occupancy probabilities across the potential habitat of red panda in Nepal (S1 Table). We extracted GIS-based landscape-level covariates for each grid cell using a data source downloaded from GIS public domain. These data include available habitat (HAB), derived bamboo cover (BAM), primary productivity—the Normalized Difference Vegetation Index (NDVI), distance to water sources (DWS), distance to nearest settlement (DNS), and elevation (ELE).

Subtropical and temperate forests constitute a broad range of habitat for red panda distribution [18]. Habitat distribution variable has direct relevance with red panda occurrence in earlier studies [11, 28]. GIS data for habitat availability (HAB), typified as the extent of temperate broadleaf forests [6] within each grid cell. Habitat data (range: 0.2–9.53 km^2^) was clipped and summarized for each grid cell (in km^2^). Bamboo cover (BAM) has been shown as a significant ground cover component for red panda habitat availability and habitat use [29]. Bamboo forms a major diet for red panda [7, 18]. We derived data for bamboo cover computed from the field-collected data on the bamboo cover. During transect walk on each grid cell, bamboo cover detections (point data) along with their respective spatial locations were recorded. We followed Wang et. al. [30] for estimating bamboo distribution whereby we used the WorldClim data [31] and field-collected bamboo points (1,856) involving MAXENT software ([32]; Fig 1 for predicted bamboo distribution). The predictive distribution model (Area under the curve (AUC) = 0.93) estimated 25,770 km^2^ of bamboo cover in Nepal (S1 File). All the layers were finally standardized using Arc Toolbox in ArcGIS 10.2. Derived GIS data on the bamboo cover were clipped and summarized for each grid cell (in km^2^) ranging between 0 to 9.53 km^2^.

NDVI is commonly used indicator to characterize vegetation primary productivity [33]. Wang et al. [34] shows coefficient of determination, expressed as R^2^, found to be significant between NDVI and Bamboo vegetation indices at 65% and 52% with or without presence of canopy in the wet season. We used Landsat 6 Thematic Mapper imagery to derive NDVI covariate [23] and images were extracted for monsoon season that matched with the timing of the 2016 survey period. GIS data characterizing the NDVI metrics (range: -0.50–0.82) were clipped and averaged for each grid cell. DWS is regarded as a major suitability factor within the red panda habitat range [7, 13, 29]. Habitat near to water sources is considered to be suitable for the red panda. In the absence of field-collected data on the sources of local running water networks (stream, rivulets and rivers), we focused on spatial layers on the river network (~ covering 194,873 km) published by the Government of Nepal’s Survey Department. We calculated the centroid for each grid cell and computed the DWS (in km) using the nearest feature vector-based analytical extension available in ArcView 3.2. DNS is used as a surrogate measure of disturbance factors at the landscape-level, which is an easily quantifiable measure that correlates with human activity [35]. We collated settlement point data (~28,459 data points) published by the Survey Department [36] to compute the distance to the nearest settlements. The Survey Department spatially defined each settlement point as clusters of households (cluster size undefined) spread in clusters across the mid-hills and high mountains. We calculated the centroid for each grid cell and computed the DNS (in km) using the nearest feature vector-based analytical extension available in ArcView 3.2. Nepal has a heterogeneous landscape with sharp elevational gradients (70–8,848 masl) within a short distance of 200 km [37]. We computed elevation (ELE) from a digital elevation model (DEM) with 90 m resolution data. We calculated an average of all the six centroids elevation points of sub-grids with each grid cell. We expected red panda occupancy and detection to be positively influenced by available habitat, bamboo cover, NDVI, the distance away from the settlement, and negatively influenced by distance to water sources and elevation. See (S1 Table) for the complete list of *apriori* hypotheses.

### Analytical design

We used the standard framework [10] to model red panda occupancy across the landscape, maximizing the likelihood of observing detection history at the sites. We used single-species, single-season occupancy models in Program PRESENCE (Version 12.7), that explicitly consider imperfect detection. We defined single season as a wet season of 2016.

All the covariates were screened for correlation [38] (S2 Table) and highly significant correlated variables (i.e. r ≥ ǀ 0.77ǀ) were either removed or not used in combination within the same model. We retained the variables that best explained the parameter of interest based on ecological relevance, corresponding to previous studies, the ease of collecting landscape level characteristics for the study areas, and the simplest in explaining the results of the model (parsimony). All covariates used in modelling were normalized using the Z transformation [22].

We used a two-stage sequential approach to model the parameter of interest at the grid level [20]. First, we modelled the influence of each of the six covariates (HAB, BAM, NDVI, DWS, DNS, AE) on the probability of detection of the red panda using the global model (the most parameterized model which included all the covariates) influencing the probability of occupancy (Ѱ). Secondly, we fixed the top model for detection and built models using different combinations of covariates influencing the probability of occupancy (Ѱ). We followed the approach of Thapa and Kelly [22] by building models for covariates influencing the parameter of interest. We modelled covariates in a stepwise, univariate fashion in such a way that if the covariate improved model fit it was retained in the model and combined with other covariates in multivariate models that we deemed important from our a priori model building. We only used combinations of covariates as additive effects in the models. We eliminated models from the candidate set that did not converge. We ranked all models using Akaike’s Information Criterion (AIC) and chose the best model based on the lowest AIC scores. We considered all models with ΔAIC < 4 as competing models [39].

Occupancy studies typically survey only a sample of grid cells [24, 40] and thereafter extends inference to the un-surveyed grid cells using covariate information from surveyed sample grid cells. We used inferences from the 446 surveyed grid cells to estimate occupancy for the 4,185 un-surveyed cells based on landscape-level covariates. We modelled site-specific probabilities of red panda occupancy as linear functions of covariates (environmental, habitat, and anthropogenic) using the logit link functions [24]. The value of untransformed coefficients (i.e. betas, β), reflects the magnitude and direction (sign) of their influence on probabilities of detection and occupancy. We considered covariates as important and supported if their respective estimates of β and the 95% confidence limits did not include zero [41]. We reported the beta estimates for the top model and univariate model. Here, we computed the model average estimates of cell specific $\hat{\Psi }$ by considering all the competing models (ΔAIC < 2 for detection model and ΔAIC < 4 for occupancy) with weightage (*w*) > 90%. We used the MacKenzie‐Bailey goodness‐of‐fit test to assess fit for most general [42]. We ran the test for 999 bootstrap iterations to generate estimates of the overdispersion factor, ĉ. If ĉ values were >1, which indicates overdispersion of data, we used AICc values adjusted for overdispersion (QAICc) [39]. We reported the final estimates on the parameter of interests (probability of occupancy and detection) for site (sampled grid cells) and landscape (for entire potential habitat) from the null (constant) model and model averaging estimates. To estimate the overall area occupied by red panda within their potential habitat, we weighed the cell-specific occupancy estimates by potential habitat available within each grid cell (9.62 km^2^) [20]. We used a parametric bootstrap [43] to compute covariance and the standard error of overall red panda landscape occupancy. We prepared predictive maps of species distribution at each unit based on inferences made from the model averaged estimates in ArcGIS 10.2. We reported the area occupied by a red panda (in km^2^) along its range in Nepal. We also reported the occupancy probabilities complex wide, PAs and outside the PAs wise within the identified complexes based on model-averaged estimates.

We evaluated the coefficient of variation (CV: standard deviation divided by the mean) [38] for each grid cell. In the final red panda occurrence prediction map, we highlighted un-surveyed cells (grid with black colour) that have covariate values far beyond the range of the surveyed cells. Site-specific variation in CV was also computed and mapped. Actual surveys were conducted at elevation range between 1,288 to 4,246 masl; hence we had little confidence in the prediction of occupancy for each grid cell that had average elevation beyond this range.

## Results

### Sampling efforts

The team walked a combined total of 1,451 km of transect walk searching for red panda sign in a total of 6,176 hours search effort and detected sign 590 times in 213 grid cells out of 446 surveyed.

### Effects of covariates on detection and occupancy

We used six covariates to model both detection probabilities, p, and occupancy, Psi (Ψ). Based on their correlation coefficients, covariates (continuous variables) were not correlated and non-significant, and therefore, were retained in the analyses (S2 Table). There was no evidence of lack-of-fit for the general model ψ suggesting AIC should be used for model selection. The overdispersion factor, ĉ was closed to 1 for both detection and occupancy general models. We compared 27 plausible a priori alternative models (11 for detection and 16 for the occupancy), which described expected combinations of the covariates influencing red panda detection and occupancy probabilities. We found the model containing the additive effect of NDVI (detection probability decreases with higher vegetation productivity) and DWS (higher detection probability as it approaches water sources) to be a top detection model (AIC = 852.49, w = 0.55, Table 1). The next competing model (AIC = 853.25, ΔAIC = 0.76, w = 0.38) containing additional covariate DNS as the second-best detection model, such that approaching settlements led to higher detection probability. The β estimate coefficients for the covariates influencing detection probabilities had varying degrees of influence and confidence intervals (CIs) did not overlap zero. This indicates support for effects of NDVI and DWS (Table 2) and confirming to our prediction expect for NDVI which was opposite. Elevation range, bamboo cover, and available habitat had no influence (ΔAIC > 15) on detection, contrary to our predictions. Although we modelled the covariates influencing Ψ using the model structure from the top detection models (NDVI + DWS) fixed, we also used the next best model (NDVI+DWS+DNS, ΔAIC = 0.76) and compared them via AIC.

**Table 1:** Effect of covariates on detection probability (*p*) of red panda across the mid-hills of Nepal.

Model	AIC	ΔAIC	*w*	Model Likelihood	K
Ψ (global), p (DWS+NDVI)	852.49	0	0.55	1	10
Ψ (global), p (DNS+NDVI+DWS)	853.25	0.76	0.38	0.6839	11
Ψ (global), p (DNS+NDVI)	856.99	4.5	0.06	0.1054	10
Ψ (global), p (DNS+DWS)	862.19	9.7	0.00	0.0078	10
Ψ (global), p (DNS)	862.47	9.98	0.00	0.0068	9
Ψ (global), p (NDVI)	863.39	10.9	0.00	0.0043	9
Ψ (global), p (DWS)	864.94	12.45	0.00	0.002	9
Ψ (global), p (ELE)	867.58	15.09	0.00	0.0005	9
Ψ (global), p (HAB)	871.65	19.16	0.00	0.0001	9
Ψ (global), p (.)	871.72	19.23	0.00	0.0001	8
Ψ (global), p(BAM)	872.8	20.31	0.00	0	9

Ψ = probability of site occupancy at the grid cell level; p = probability of detection; AIC is Akaike's information criterion, ΔAIC is the difference in AIC value of the focal model and the best AIC model in the set, K is the number of model parameters and –2Loglik is -2 of the logarithms of the likelihood function evaluated at the maximum. Covariates considered: DNS: Distance to nearest settlement; DWS: distance to nearest water sources; NDVI: Normalized differential vegetation index; ELE: Average elevation, HAB: Available habitat, BAM: Bamboo cover in each grid cell, # In all models the probability of occupancy (Psi) was modelled as “Ψ” (global: ELE+NDVI+NDS+HAB+BAM+DWS), ‘+’ denotes covariates modelled additively.

**Table 2:** β estimates and standard errors [in parentheses] from the logit link function based on the best and the univariate, single-species, single-season occupancy models for red panda detection probability (p) in mid-hills and high Himalayas in 2016.

Model	Intercept	${\hat{\beta }}_{AE}$ $\left(SE[\hat{{\hat{\beta }}_{ELE}}]\right)$	${\hat{\beta }}_{DWS}$ $\left(SE[\hat{{\hat{\beta }}_{DSW}}]\right)$	${\hat{\beta }}_{HAB}$ $\left(SE[\hat{{\hat{\beta }}_{HAB}}]\right)$	${\hat{\beta }}_{BAM}$ $\left(SE[\hat{{\hat{\beta }}_{BAM}}]\right)$	${\hat{\beta }}_{DNS}$ $\left(SE[\hat{{\hat{\beta }}_{DNS}}]\right)$	${\hat{\beta }}_{NDVI}$ $\left(S\hat{E}[{\hat{\beta }}_{NDVI}]\right)$
A *priori* relationship		-	-	+	+	+	**+**
Best Model (*w* = 0.55)	0.62(0.20)		**-0.47(0.12)**				***-0*.*68(0*.*19)***
Second Best Model (*w* = 0.38)			**-0.35(0.15)**			0.53(0.44)	**-0.64(0.20)**
Univariate Model		***0*.*83(0*.*35)***	**-0.35(0.11)**	-0.32(0.22)	-0.81(0.69)	**1.16(0.36)**	**-0.62(0.20)**

ELE: Average elevation; DWS: distance to nearest water sources; HAB: Available habitat; BAM: Bamboo cover in each grid cell; DNS: Distance to nearest settlement; NDVI: Normalized differential vegetation Index;, # In all models the probability of occupancy (Psi) was modelled as “Ψ” (global: ELE+NDVI+NDS+HAB+BAM+DWS), ‘+’ denotes covariates modelled additively. *w* = model weight SE: standard error. Bold indicates strong or robust impact, that is 95% confidence intervals as defined by $\hat{\beta }\pm 1.96\times \mathrm{SE}$ not overlapping at 0; *Italics* indicate opposite from *a priori* prediction.

**Fig 2:**
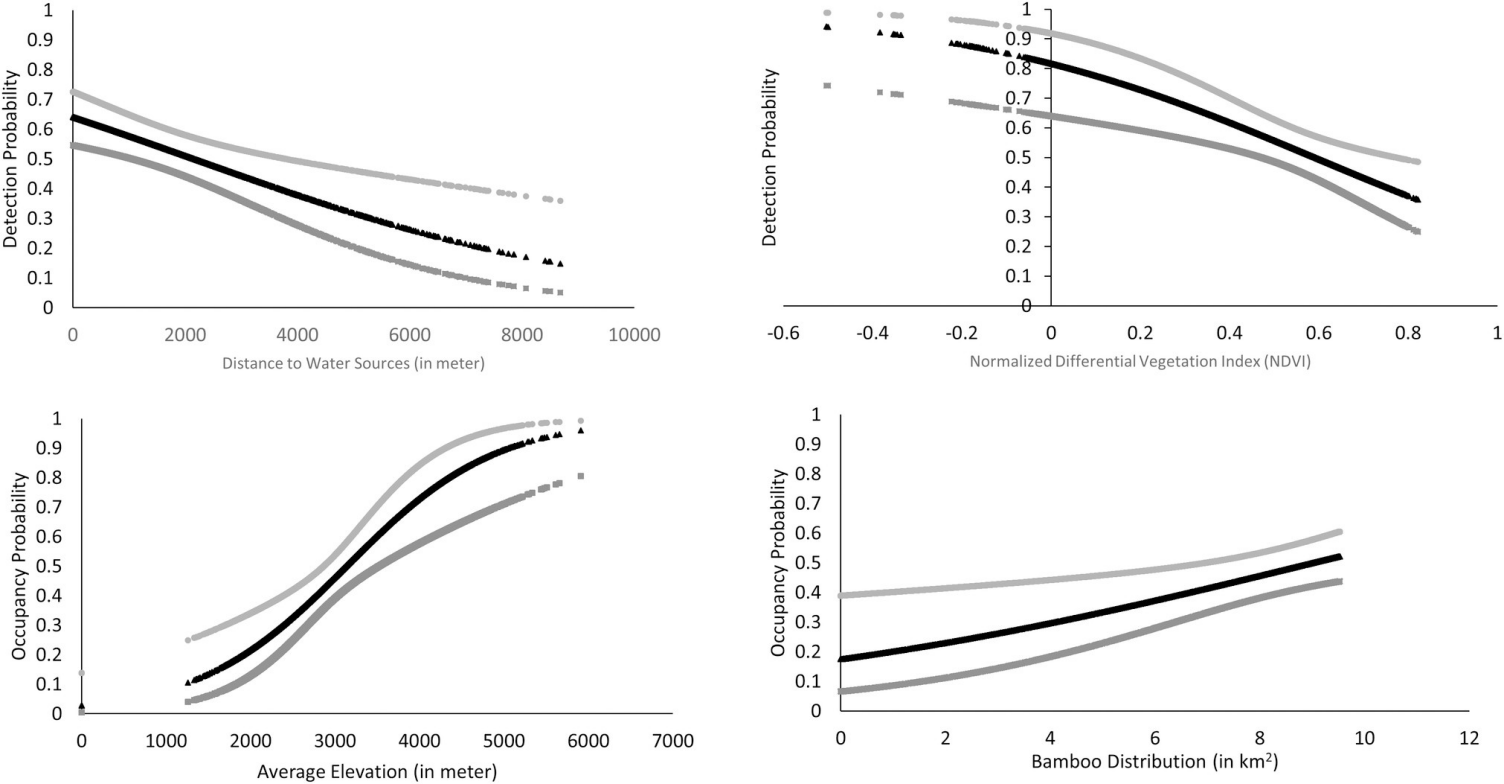
Relationships between highly influential covariates based on beta estimates (β) from univariate models and the probability of red panda detection (top) and occupancy (bottom).

Inclusion of the second-best detection model (NDVI+DWS+DNS, ΔAIC = 21.28) did not change the top model influencing occupancy (ELE+BAM+DWS, *w* = 0.81) which contains the top detection model (NDVI+DWS) (Table 3). We also found a model containing additive effect of the covariate of ELE+DWS (AIC = 853.2, ΔAIC = 3.99, *w* = 0.11) to be the next competing model. Comparison of beta estimates from competing models (less than 4ΔAIC, *w* = 92%) indicated that elevation (ELE), bamboo cover (BAM) and distance to water sources (DWS) have a significant (non-overlapping CIs at zero) influence indicating support for occupancy (Fig 2). However, an expectation of ELE and DWS was opposite while BAM was in concordance with our *apriori* predictions (Table 4).

**Table 3:** Effect of covariates on occupancy (Ψ) of red panda across the mid-hills of Nepal.

Model	AIC	ΔAIC	*w*	Model Likelihood	K
Ψ (ELE+BAM +DWS), p (DWS+NDVI)	849.21	0	0.81	1.00	7
Ψ (ELE+DWS), p (DWS+NDVI)	853.2	3.99	0.11	0.14	6
Ψ (ELE+BAM), p (DWS+NDVI)	854.57	5.36	0.06	0.07	6
Ψ (ELE+BAM+DNS), p (DWS+NDVI)	856.33	7.12	0.02	0.03	7
Ψ (ELE), p (DWS+NDVI)	861.78	12.57	0.00	0.00	5
Ψ (ELE+DNS), p (DWS+NDVI)	863.49	14.28	0.00	0.00	6
Ψ (BAM+DNS), p (DWS+NDVI)	867.77	18.56	0.00	0.00	6
Ψ (BAM+DWS), p (DWS+NDVI)	869.19	19.98	0.00	0.00	6
*Ψ (*.*)*, *p (DWS+NDVI+DNS)*	870.49	21.28	0.00	0.00	5
Ψ (BAM), p (DWS+NDVI)	871.78	22.57	0.00	0.00	5
Ψ (DWS), p (DWS+NDVI)	873.61	24.4	0.00	0.00	5
Ψ (DNS), p (DWS+NDVI)	874.43	25.22	0.00	0.00	5
Ψ (HAB), p (DWS+NDVI)	874.63	25.42	0.00	0.00	5
Ψ (NDVI), p (DWS+NDVI)	875.67	26.46	0.00	0.00	5
Ψ (.), p (DWS+NDVI)	877.57	28.36	0.00	0.00	4
Ψ (.), p (.)	900.13	50.92	0.00	0.00	2

Ψ = probability of site occupancy at the grid cell level; p = probability of detection; AIC is Akaike's information criterion, ΔAIC is the difference in AIC value of the focal model and the best AIC model in the set, K is the number of model parameters and –2Loglik is -2 of the logarithms of the likelihood function evaluated at the maximum. Covariates considered: DNS: Distance to nearest settlement; DWS: distance to nearest water sources; NDVI: Normalized Differential Vegetation Index; ELE: Average Elevation, Hab: Available Habitat, BAM: Bamboo cover in each grid cell, # In all models the probability of detection was modelled as “p” (DNS+NDVI)”. ‘+’ denotes covariates modelled additively. *Ψ (*.*)*, *p (DWS+NDVI+DNS)*: inclusion of second-best detection model.

**Table 4:** β estimates from the logit link function based on best and univariate models for red panda occupancy probability (Psi).

Model	Intercept	${\hat{\beta }}_{AE}\;(S\hat{E}[{\hat{\beta }}_{ELE}])$	${\hat{\beta }}_{NDVI}\;(S\hat{E}[{\hat{\beta }}_{NDVI}])$	${\hat{\beta }}_{HAB}\;(S\hat{E}[{\hat{\beta }}_{HAB}])$	${\hat{\beta }}_{BAM}\;(S\hat{E}[{\hat{\beta }}_{BAM}])$	${\hat{\beta }}_{DNS}\;(S\hat{E}[{\hat{\beta }}_{DNS}])$	${\hat{\beta }}_{DWS}$ $\left(S\hat{E}[{\hat{\beta }}_{DWS}]\right)$
A *priori* relationship		-	+	+	+	+	-
Best Model (*w =* 0.81*)*	-0.46 (0.33)	***1*.*22(0*.*30)***			****0.75(0.30)****		***0*.*57(0*.*27)***
Univariate Model		***0*.*98(0*.*25)***	*-0*.*37(0*.*19)*	*-0*.*45(0*.*47)*	****0.67(0.25)****	****0.68(0.31)****	*0*.*43(0*.*22)*

Covariates considered: ELE: Average elevation; NDVI: Normalized differential vegetation index; HAB: Available habitat; BAM: Bamboo cover in each grid cell; DNS: Distance to nearest settlement; DWS: distance to nearest water sources. Bold indicates strong or robust impact, that is 95% confidence intervals as defined by $\hat{\beta }\pm 1.96\times \mathrm{SE}$ not overlapping at 0; Italics indicate opposite from a priori prediction. Figures in the parenthesis denotes standard error (SE).

### Static estimates of detection and occupancy probabilities

**Fig 3:**
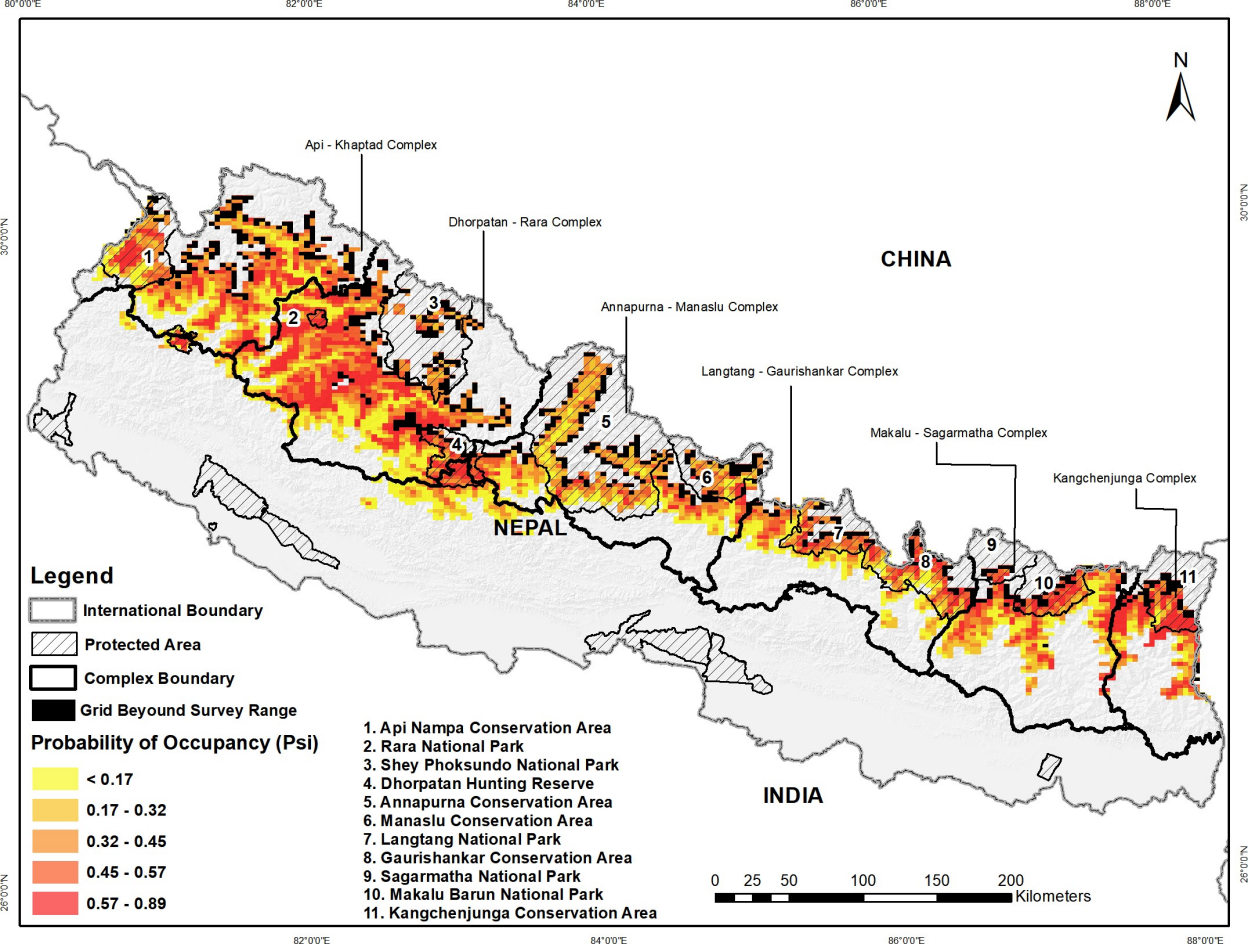
Site specific variation in red panda occupancy based on top model along the mid-hills and high mountains of Nepal. Grid shading (with darker red color indicating higher probability of occupancy) shows site specific occupancy probabilities in the wet season of 2016 using single-species, single-season occupancy model. Grid with dark color shows area beyond the survey range.

**Fig 4:**
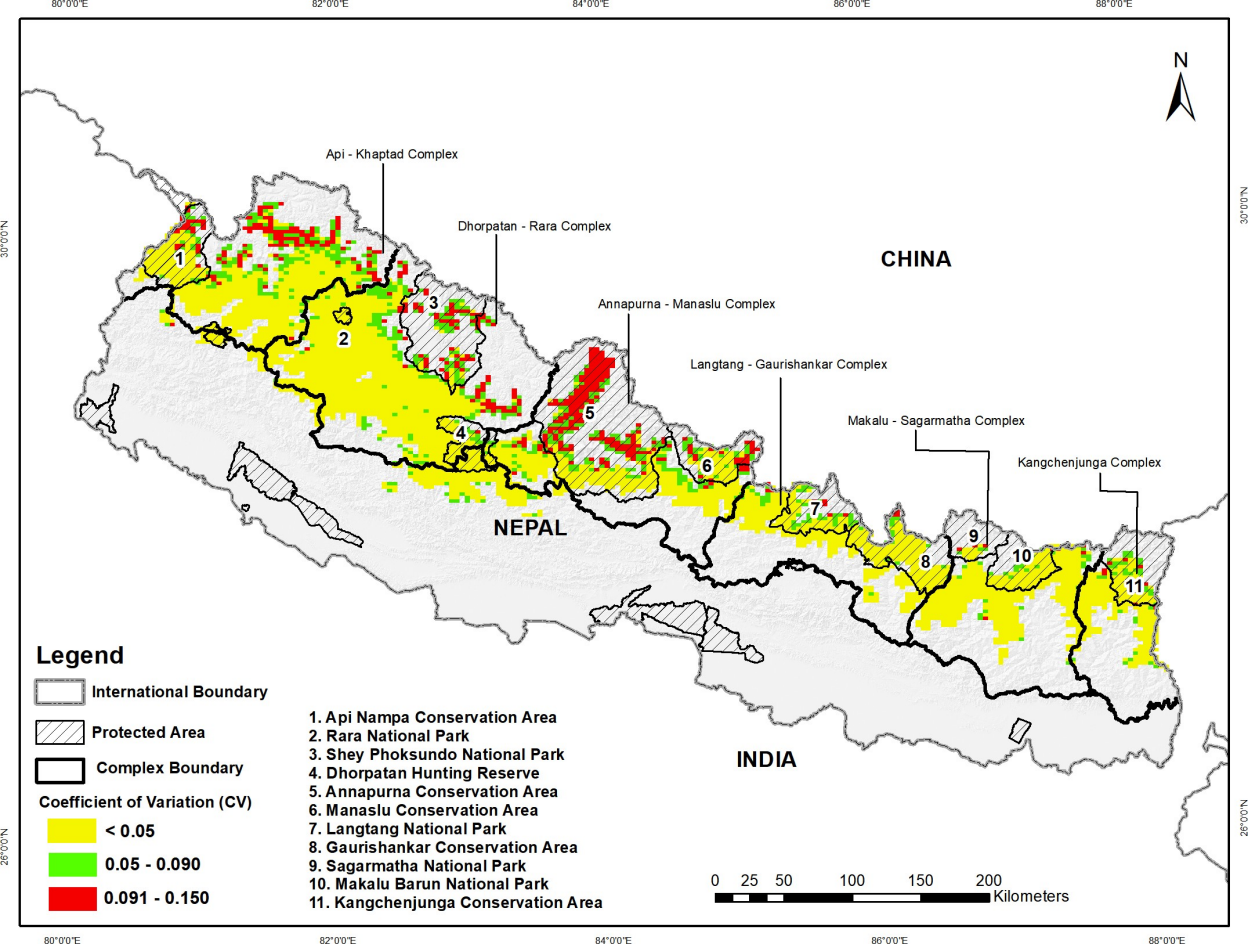
Variation (expressed in CV: Coefficient of variation, in %) in occupancy pattern based on estimated probabilities of occupancy in the wet season of 2016 using single-species, single-season occupancy model.

Estimates of red panda detection probability ($\hat{p}(SE(\hat{p}))$, based on top model) while walking on transects was 0.70(0.02). Naïve occupancy, which fails to account for imperfect detection, was estimated at 0.32. Null model occupancy was estimated at 0.47(0.04). Red panda site occupancy ($\hat{\Psi }(SE(\hat{\Psi }))$ along the 446 surveyed cells was estimated at 0.48(0.01). The final estimates of red panda landscape occupancy for Nepal, including projections for non-surveyed grid cells (*w* = 0.92, ΔAIC) was 0.40(0.02). We estimated the site-specific $\hat{\Psi }$ using the model averaging estimates and showed the matrix of site-specific variation addressing the uncertainties in occupancy (within elevation range: 1,288–4,246 masl) across the geographical space in the country (Fig 3). Site-specific occupancy probabilities CVs (in %) was averaged at 3.63 with a range between 0.67 (minimum) and 14.1 (maximum) (Fig 4). During the survey period, the area of potential habitat occupied by red panda was 10,151 km^2^ out of the total 22,453 km^2^ of potential habitat available in the country.

We estimated the highest occupancy with 0.52(0.08) (3,730 km^2^; SE  = 573 km^2^) of the 7,173 km^2^ of potential habitat occupied by a red panda in the Kangchenjunga complex. In contrast, lowest occupancy was estimated at 0.35(0.08) (3,798 km^2^; SE  =  868 km^2^) of the 10,853 km^2^ of available habitat occupied in the Langtang-Gaurishankar complex (Table 5). PAs system harbouring the red panda potential habitat collectively has the highest occupancy at 0.45(0.11) than the area outside the PAs at 0.40(0.08).

**Table 5:** Comparison of red panda occupancy estimates (Ψ, SE(Ψ)) based on top model in six conservation complexes, protected areas, and outside protected area of Nepal [6].

****Red Panda Conservation Complex****	****Complex wise Occupancy****	****Protected Areas Occupancy****	****Outside Protected Areas Occupancy****
	Ψ SE(Ψ)	Ψ SE(Ψ)	Ψ SE(Ψ)
Kangchenjunga	0.52(0.08)	0.60(0.11)	0.49(0.07)
Makalu-Sagarmatha	0.40(0.07)	0.51(0.10)	0.37(0.06)
Langtang-Gaurishankar	0.35(0.08)	0.44(0.09)	0.27(0.06)
Annapurna-Manaslu	0.38(0.10)	0.40(0.12)	0.35(0.08)
Dhorpatan-Rara	0.45(0.08)	0.50(0.12)	0.44(0.07)
Api-Khaptad	0.40(0.09)	0.39(0.08)	0.40(0.10)

## Discussion

The conservation status of the iconic red panda is a measure of regional conservation efforts in the mid-hills and high mountains. We utilized robust single-species, single-season occupancy framework [44], first of its kind which has been tested for the large-scale surveys [21, 45], producing reliable estimates for quantifying the distribution status of the red panda. Our study provides the first estimates of detectability and site occupancy for a red panda in their range and is a crucial first step in monitoring seldom seen arboreal species, many of which are suspected to be declining and occupy critical habitats. Major findings have been 1) red panda occupancy was estimated at 0.40 and serves as a baseline for this important arboreal species, 2) distance to water sources and normalized differential vegetation index was found to be influencing the red panda detection probability, 3) occupancy of the red panda was strongly influenced by elevation, distance to water sources, and the bamboo cover, and 4) site-specific variation was observed in occupancy probability (CV:0.67–14.10; Av.CV:3.63) along the proposed red panda conservation complex.

Red panda varied substantially in site occupancy (0.00–0. 90). Our analysis addressed the uncertainties through inclusion of data within the recorded elevational range and model averaging estimates (with low variance) best predicts the baseline estimate for the red panda occupancy. Our findings show the hotspots (sites with a higher probability of occupancy, Fig 3) for effective red panda conservation and also corresponds with an earlier study that advocated for the creation of special red panda conservation zones [9], perhaps within the conservation complex.

Our modelling approach incorporating imperfect detection with probabilistic model and including a priori hypothesis generated robust results that could be interpreted. Thus, this study is first of its kind that addresses the issue of detectability (~estimated at 0.70) in estimating the occupancy (~ estimated at 0.40) for the arboreal species at a large scale. Our site-specific estimates on red panda occupancy and predictive mapping provide managers (PAs and Division Forest Office) with required estimates necessary for spatial planning and rolling out specific management actions such as habitat management, protection measures for securing red panda and their habitat including fine scale population assessment of red panda. This serves as an opportunity for the objectively defining the sources sites for targeting conservation efforts, as done in protected area level assessment for a red panda in Dhorpatan Hunting Reserve in Nepal [12], and dhole in Bandipur reserve in India [46]. Our assessment on red panda occupancy probabilities at the protected area level suggest higher estimates (Psi = 0.45) than the area outside the protected areas (Psi = 0.40). The majority of the potential red panda habitat lies within mid-hills. PAs coverage in mid-hills of Nepal is only 1.33% [47] thus marking a small difference in occupancy probabilities when compared with an area outside the PAs. Kalikot district in Dhorpatan-Rara Conservation Complex limits the westernmost distribution of species (~ true presence). However, our result estimated occupancy probability at 0.40 in Api Khaptad Conservation complex located west of Kalikot district. In the past, locals in Api Nampa Conservation Area (westernmost PAs in Nepal, around Ghusa and Khandeswori) have reported sightings of red pandas however this information lacks verification from PA authorities (Personal Communication: Ashok Ram, former warden, Api Nampa Conservation Area). However, in the past, PA officials have apprehended poacher with red panda skins in the region. The complex-wide distribution of red panda provides an objectively defined baseline assessment for zoning complexes for red panda conservation. Within each conservation complex, multiple modes of management exists such as protected areas and national forest including the community forest program [48]. The custodian of communities under the community forest user groups provides an opportunity for inclusion of red panda conservation and its management actions in their forest operation plan.

We estimated around ~10,151 km^2^ of the total potential habitat is occupied by the red panda in Nepal. Previous results shows that the predicted habitat distribution ranged between ~592 km^2^ [6], 8,200 km^2^ [49], 21,680 km^2^ [50] to 17,400 km^2^ to 22,400 km^2^ [51]. These studies have estimated potential distribution as an index of suitability; hence our results were non-comparable due to methodological differences as the former studies are deeply rooted in the presence and absence approach.

We developed a distribution (detection and occupancy) models for a red panda in Nepal and landscape variables influencing it. The model weight was concentrated on the most-favored covariates for detectability (two) and covariates for occupancy (three). Distance to water sources and normalized differential vegetation index shows a strong influence (95% CI did not overlap 0) on red panda detection probability. Supporting our *apriori*, detection probability tends to increase with a decrease in distances to water sources. Landscape variable defined here and used in modelling detection probability is the major river network found across the range. While field level covariates comprised of all perennial and seasonal tributaries observed during the fieldwork conducted in the wet seasons. Although there were differences in scale (landscape versus field), our result was similar to previous studies where 70% of red panda signs were located within a range of 0 to 100 m from water sources [11, 18, 52]. Our survey conducted in the wet season facilitated the detection of red panda signs (with high detection probability observed here) thus it can be argued that water availability was not a limiting factor in the monsoon season. But high decay rate of red panda faeces and washing of red panda sign in rainy season confirms that detectability could be even high in wet season than reported here. To expand the survey in the future incorporating multi-season detection data, the season could be one of the deterministic factors influencing red panda detectability. Precipitation level varies significantly between winter and summer as compared to the monsoon season [53]. We found negative influence of NDVI on red panda detectability as probability tends to decrease with an increase in NDVI. Bamboo as an understory and conifer forest canopy are contributing towards the red panda habitat. From start to the end of the wet season, NDVI usually becomes maximum (greenness) at end of the season. Zhang et al. [54] argued that greenness is insensitive to droughts but more related to radiation during the wet season. Thus, low structural response of habitat with more open tree canopies might be contributing low productivity influencing high detection. Bista et al. [55] found a high preference of red panda with a high tree with low canopy cover especially in the eastern part of the country. This could be attributed to their adaptation to conserve energy and thermoregulation by ensuring maximum exposure to the sun in temperate habitat.

Bamboo cover, elevation, and distance to water sources appeared in the top two models (cumulative weightage, *cw* ~92%) for occupancy and appeared to be important determinants of red panda distribution. Supporting our *apriori*, bamboo cover positively influences red panda occupancy with strong effect (95% CI did not overlap 0) and our finding is comparable to previous results [52, 56–59] where the availability of bamboo cover was identified as a significant predictor. The major contribution of the red panda diet comprised of Bamboo [13, 28] where leaves and shoots constitute 83% of the overall diet of red pandas [18]. The bamboo cover is estimated at 25,770 km^2^ spread around entire potential red panda habitat as ground cover (Fig 1). Conservation of this floral species is imperative to red panda conservation. Floral conservation action plan prepared for floral species such as Bijaysal (*Pterocarpus marsupium*) [60] and Rhododendron (*Rhododendron spp*.) [61] can be replicated for bamboo. Prioritized conservation actions can bring indirect relevance with improving the red panda occupancy in the complex as observed in this study. Thus, the inclusion of Bamboo cover in defining the site occupancy does corroborate with earlier studies [5, 52, 55]. Opposite to our *apriori*, an increase in elevation range found to be positively influencing the red panda occupancy with a significant effect. However, result needs to be interpreted with caution. In this survey, we recorded red panda presence between 2,361–4,246 masl. More than 75% of the red panda detections were recorded in grid cells with average elevational range between 1,930–3,850 masl and clustering maximized at grid cells between average elevational range 2,600–2,900 masl. Our results are similar to Pradhan et al. [52] who found a high occurrence of red panda in a narrow range between 2,800–3,100 masl in Singalila National Park, India. These elevation ranges are regarded as highly preferred to moderately preferred habitat for red panda [11, 12]. Predicted occupancy estimates also overlap with earlier distributional range i.e. between 2,200–4,800 masl defined for a red panda [14]. Elevation together with slope, aspect and seasons tends to bring in humid climatic conditions influencing the habitat conditions [62, 63] (tree and shrubs, tree structure) that is preferable to the red panda. Humid climate condition could influence the ambient temperature that could influences the habitat condition such as suitability of bamboo cover (S1 File) including the metabolism of the species [64]. Our results incorporating the landscape covariates and similar studies ([11, 50, 55]) incorporating the field level covariates influencing red panda fine-scale habitat selection and their distribution along the range were found to be complementing each other. Distance to waters sources had a positive influence on occupancy but opposite to our expectation such that high occupancy with increase in distance from water sources. Previous studies based on field level covariates on water sources as mentioned earlier suggested that red panda has high affinity to water sources but avoiding the larger river network comprising of both seasonal and perennial river sources. Habitat away from water sources influenced the red panda presence that also contributed towards the variation in red panda abundance and affecting detection probability as well [65].

Our understanding of covariate relationships with the focal parameter of interest could be improved by increasing sample size (i.e., grid cells) along the environmental gradient. This will also allow using field-level covariates in occupancy modelling. Use of field level covariates such as done in Dhorpatan, Nepal for red panda [12], tigers and their prey in Churia habitat [22], and dhole in Bandipur Tiger Reserve, India [46] will guide to determine the fine-scale ecological determinants to red panda distribution. This is particularly crucial for site-level assessment in predictive zones with high probabilities of site occupancy. Habitat degradation is an imminent threat to red panda conservation. We used proxy disturbance covariate such distance to nearest settlements but did not find it as competing model. Large infrastructure projects such as district and local roads, North-South highways under belt and road initiative, hydel transmission lines, and dams are rapidly developing along the identified red panda complex and should be explored for their disturbance effect on red panda population and habitat.

The results of our occupancy analysis can act as a baseline to measure where and how these factors affect red panda distribution and occupancy in the future (given the landscape covariates we have suggested). Estimating the population size is a very expensive proposition at such a large scale, hence occupancy modelling is a suitable option [66]. Since it is based on detection and non-detection data, it is a suitable proposition, cheaper and more robust approach to quantify distribution and factors affecting it [20, 66]. In absence of camera trapping, especially for unmarked species, occupancy employing landscape variables provides analytical avenue for large scale sign-based surveys. Baseline provides an opportunity to detect changes in occupancy over time which has been rarely tested in large spatial scales for red panda. Monitoring changes in occupancy including abundance over time in documenting changes in conservation status [67] should be priority initiatives along the range and special zones in PAs or in areas outside the PAs within an identified complexes. The role of local citizen scientists has been crucial in the present survey especially in the collection of required information from red panda habitat in their surroundings. Future monitoring incorporating a robust design framework with the use of citizen scientist seems promising [27]. Government of Nepal’s five years periodic Red Panda Conservation Action Plan (2019–2023) [68] prioritizes conservation actions to protect and manage red panda populations in Nepal. Updating National population status and occupancy-based distribution regularly (at least every five years) is recommended as prioritized action, and replication of the analytical techniques used in this study will help to update red panda occupancy nationwide.

## Supporting information

S1 File Methodology for deriving the bamboo distribution in Nepal. (PDF)

S1 Table Landscape-level predictor variables (including their justification) used as potential factors influencing red panda detection and occupancy. The “+” and “-” indicates the *a priori* predictions regarding the hypothesized direction of the effect.
(DOCX)

S2 Table Spearman correlation coefficients between the predictor variables. (DOCX)
