# Supplementary material for: Landscape variables affecting the Himalayan red panda Ailurus fulgens occupancy in wet season along the mountains in Nepal
Source: PLoS One. 2020 Dec 11;15(12):e0243450. doi: 10.1371/journal.pone.0243450 (PMC7740865; doi:10.1371/journal.pone.0243450)
Supplement: S1 File — (PDF) [file pone.0243450.s001.pdf]

S1 File

Methodology for deriving the Bamboo distribution in Nepal

Himalayan temperate broadleaved forest with bamboo in the understory are found with an altitudinal range preference of 2400-3900 meter (Yonzon and Hunter, 1991). Red panda prefers to live in forests close to water sources (within 100-200m) and with moderate tree canopy (>30%) and bamboo cover (>37%) where an average bamboo height should be more than 2.9 meter (Yonzon and Hunter, 1991; Pradhan et al., 2001; Williams 2006; Dorji et al., 2012).

The distribution of bamboo in Nepal is estimated at 25,770 sq. km. which are spread over 53 districts of Nepal. The overall elevation of Bamboo distribution ranges from 938 to 5,917 meter. The national survey of red panda considered the presence of bamboo as a potential habitat for red panda and selection of sample site for the survey.

Methods:

1. The vegetation analysis in terms of Importance Value Index (IVI) of trees; and bamboo cover, their height and abundance were also considered.

2. During the survey altogether 160 herbariums of bamboo from different areas of identified sampling sites were also collected during this survey which has been stored, catalogued, and arranged systematically for further identification.

3. Based on the previously available red panda presence data and environmental parameters including 19 bioclimatic variables along with altitude, slope, aspect, land cover and Normalized Difference Vegetation Index (NDVI), MaxEnt Modeling using MaxEnt version 3.3.3k was done.

4. The Predictive distribution model developed in MaxEnt at the Area under Curve (AUC) value of 0.926).

5. Thus, identified habitat was overlaid with grids (9.6 Km^2) and the grids which cover nearly 50% area of the identified habitat were considered for random selection upto 50% of the total grids.
6. Each of the selected grid was further overlaid with 6 sub-grids (area = 1.6 km^2), and again the 3 of them were randomly selected as sampling site. In this way, a total number of 856 sub-grids were selected as sampling sites for occupancy survey (GoN/MoFSc, 2016).

Altogether 17 species of bamboo (Table 1) have been documented during red panda survey across red panda range from all 35 districts. During field survey *Drepanostachyum* spp. of bamboo is documented most often and *Borindachigar* least often. The bamboos were identified on the basis of Manual of Bamboos of Nepal (TIS, 2014) and Bamboos of Nepal (Stepleton, 1994).

SN	Local Name	Scientific Name	Location	Ecological Range	Habitat
1	Sano Maling	*Arundinaria racemosa*	Manang, Nuwakot, Okhaldhunga, Ramechap, Solukhumbu,	2600-3500m	High mountain forests of Oaks, Abies, fir and spruce.

SN	Local Name	Scientific Name	Location	Ecological Range	Habitat
2	Chigar	*Borindachigar*	Jumla, Kalikot, Nuwakot, Rolpa, Solukhumbhu, Sinduplalchok, Sankhuwasaba	2600-3100m	High mountain forests composed of Abies, fir and rhododendrons.
3	Kalo Nigalo	*Borindaemeryi*	Nuwakot, Sindupalchok, Sakhuwasaba	2600-3200m	High mountain forests composed of Abies, fir and rhododendron forests in eastern Nepal.
4	Diu/Tite Nigalo	*Drepanostachyum falcatum*	Achham, Bajura, Jajarkot	1000-2000m	Evergreen forests of *Schima walichii*, Castanopsis forests and occasionally in chirpine forest.
5	Tite Nigalo	*Drepanostachyum falcatum* var. *glomeratum*	Jajarkot, Bajura, Humla, Nuwakot, Sankhuwasaba, Dolpa	1000-2000m	*Alnus nepalensis/Pine/rhododendron* forest.
6	Tite Nigalo	*D. intermedium*	Jajarkot, Bajura, Humla, Nuwakot, Sankhuwasaba, Dolpa	1000-2000m	Oak and Chestnuts forest also in drier subtropical forests.
7	Ban Nigalo	*D. khasianum*	Jajarkot, Bajura, Humla, Nuwakot, Sankhuwasaba, Dolpa	1000-2000m	Oaks, *Schima walichii* and chestnut forests.
8	Putru /Putre/ Suruwal Nigalo	*Drepanostachyum* spp.	Jajarkot, Bajura, Humla, Nuwakot, Sankhuwasaba, Dolpa	1500-2300m	*Alnus nepalensis and* Rhododendron forest, also cultivated at well drained moist sites.
9	Ghunre Nigalo, Malinge Nigalo	*Himalayacalamus asper*	Manang, Lamjung	1800-2300m	Oak, rhododendron and castonopsis forests.
10	Malinge, Nigalo	*H. brevinodus*	Bajura, Humla	1800-2200m	Oak, rhododendron and Castanopsis forests.
11	Malinge, Nigalo	*H. cupresus*	Manang, Lamjung	2300-2800m	Oaks, rhododendrons and Castanopsis forests.
12	Thudi Nigalo, Singhane	*H. falconerii*	Manang	2000-2500m	Oaks, rhododendrons and Castanopsis forests.
13	Tite Nigalo	*Himalaya calamus fimbriatus*	Dolpa	1100-2000m	Oaks, rhododendrons and Castanopsis forests.

SN	Local Name	Scientific Name	Location	Ecological Range	Habitat
14	Padang	*Himalaya calamus hookerianus*	Humla	2000-2500m	Prefers well drained moist sites and grows well under moderate shade of Alnus, rhododendrons and oaks.
15	Seto Nigalo	*Himalaya calamusporcatus*	Dolpa	2000-2300m	Oaks, rhododendrons and Castanopsis forests.
16	Malingo Nigalo	*Himalaya calamus sp.*	Kalikot, Lamjung, Okhaldhunga, Manang	2300-2700m	Oaks and rhododendrons.
17	Lahure Bans, Nigale Bans	*Melocanna bacifera*	Lamjung, Manang	Terai-1400m	Prefers well drained moist sites and reported to be grown well in mixed sal forests of Bangladesh and Northeastern India.

Reference

Dorji, S., Rajaratnam, R., & Vernes, K. (2012). The Vulnerable red panda *Ailurus fulgens* in Bhutan: distribution, conservation status and management recommendations. Oryx, 46(04), 536-543.

Dorji, S., Rajaratnam, R., & Vernes, K. (2012). The Vulnerable red panda *Ailurus fulgens* in Bhutan: distribution, conservation status and management recommendations. Oryx, 46(04), 536-543.

GoN/MoFSc, 2016. National Survey of Red Panda, Ministry of Forests and Soil Conservation, Government of Nepal, Singha Durbar, Kathmandu, Nepal

Pradhan, S., Saha, G. K., & Khan, J. A. (2001). Ecology of the red panda Ailurus fulgens in the Singhalila National Park, Darjeeling, India. Biological Conservation, 98(1), 11-18.

Stapleton, C. M. A. (1994). The bamboos of Nepal and Bhutan. Part I: *Bambusa, Dendrocalamus, Melocanna, Cephalostachyum, Teinostachyum, and Pseudostachyum* (Gramineae: Poaceae, Bambusoideae). Edinburgh Journal of Botany, 51(01), 1-32.

TIS. 2004. Manual on Bamboos of Nepal. Tree Improvement and Silviculture (TIS), Natural Resources Management Sector Assistance Programme (NARMSAP), Department of Forests, Series No. 107. Kathmandu, Nepal.

Williams, B.H. (2006). Red panda in Eastern Nepal: How does it fit into Eco-regional Conservation of the Eastern Himalaya? In: J.T. McCarthy (Eds.), Conservation Biology in Asia. Society of Conservation Biology and Resources Himalaya, Kathmandu, Nepal, pp. 236-251.

Yonzon, P. & M .L. Hunter 1991a. Conservation of the Red Panda (Ailurus fulgens), Biological. Conservation. 57(1991) 1-11.
