# Supplementary material for: Landscape variables affecting the Himalayan red panda Ailurus fulgens occupancy in wet season along the mountains in Nepal
Source: PLoS One. 2020 Dec 11;15(12):e0243450. doi: 10.1371/journal.pone.0243450 (PMC7740865; doi:10.1371/journal.pone.0243450)
Supplement: S1 Table — The “+” and “-” indicates the a priori predictions regarding the hypothesized direction of the effect. (DOCX) [file pone.0243450.s002.docx]

S1 Table. Landscape-level predictor variables (including their justification) used as potential factors influencing red panda detection & occupancy. The “+” and “-” indicates the apriori predictions regarding the hypothesized direction of the effect.

| **Covariates** | **Justification for the selection of the covariates** | **Description** | **Value Range** | | | **Hypothesized apriori relationship to red panda occupancy probability** |
| --- | --- | --- | --- | --- | --- | --- |
|  |  |  | **Min** | **Max** | **Av. (SD)** |  |
| Distance to water sources  (DWS) (in km) | Red panda detection tends to increase with decrease in distance to water sources | Digitized data on major river network derived from Survey Department, Government of Nepal. | 0.00 | 8.68 | 1.41 (1.29) | + |
| Distance to Nearest Settlement (DNS) (in km) | Red panda detection and occupancy tends to increase with increase in distance from the settlement areas (proxy to disturbance) (Matyukhina *et. al* 2014) | Generated a surface by calculating the Euclidean distance from settlement data extracted from Nepal Survey Department 1996 digital topographic data and world settlement data. | 0.06 | 23.16 | 2.35 (2.72) | + |
| Normalized Difference Vegetation Index (NDVI) | Red panda detection and occupancy tends to increase with increase in vegetation productivity. | Derived from Modis satellite imagery (250 m X 250 m resolution) of the study area during the survey season (July-September 2016). Download from : <http://modis.usgs.gov> | -0.50 | 0.82 | 0.39 (0.24) | + |
| Elevation  (ELE) (in m) | Red panda habitat lies between 1500 to 4500 m (Jnawali *et. al* 2011) thus there is high tendency to decrease in detection & occupancy during the range. | Derived from Digital Elevation Model/DEM at 90-m resolution from the Shuttle Radar Topographic Mission dataset (SRTM) (Jarvis et al. 2008); Downloaded from: <http://srtm.usgs.gov> | 1258 | 5910 | 3203(862) | - |
| Habitat Available (HAB) (km^2^) | Habitat availability is the amount of total habitat available in the area. Higher the availability higher detection & occupancy of red panda. | Derived and extracted for the study area from 2010 supervised classification Landsat 6 Thematic Mapper imagery (28.5 m X 28.5 m resolution) with permission from WWF Nepal. Download from : <http://Glovis.usgs.gov> | 0.02 | 9.53 | 9.44 (0.70) | + |
| Bamboo cover (BAM) | Bamboo cover is important as it provides the shade and dictates the kind of ground cover (Thapa *et. al* 2018). Higher the bamboo cover, higher the red panda detection & occupancy. | Derived and extracted for the study area from 2011 supervised classification of Landsat 4–5 Thematic Mapper imagery (30 m X 30 m resolution) with permission from WWF Nepal. Using Maxent to derive the Bamboo distribution. | 0.00 | 9.53 | 5.07 (3.89) | + |

Min: Minimum; Max: Maximum; Av.: Average; SD: Standard Deviation
