# Supplementary material for: Landscape variables affecting the Himalayan red panda Ailurus fulgens occupancy in wet season along the mountains in Nepal
Source: PLoS One. 2020 Dec 11;15(12):e0243450. doi: 10.1371/journal.pone.0243450 (PMC7740865; doi:10.1371/journal.pone.0243450)
Supplement: S2 Table — (DOCX) [file pone.0243450.s003.docx]

S2 Table. Spearman correlation coefficients between the predictor variables.

| **Covariates** | **ELE** | **NDVI** | **DNS** | **HAB** | **BAM** | **DWS** |
| --- | --- | --- | --- | --- | --- | --- |
| **ELE** | 1.00 |  |  |  |  |  |
| **NDVI** | -0.72 | 1.00 |  |  |  |  |
| **DNS** | 0.63 | -0.58 | 1.00 |  |  |  |
| **HAB** | -0.02 | 0.02 | -0.04 | 1.00 |  |  |
| **BAM** | -0.39 | 0.60 | -0.36 | 0.01 | 1.00 |  |
| **DWS** | 0.12 | -0.01 | 0.06 | 0.02 | 0.15 | 1.00 |

Covariates considered: ELE: Average elevation; NDVI: Normalized differential vegetation index; DNS: Distance to nearest settlement; HAB: Available habitat; BAM: Bamboo cover in each grid cell; DWS: distance to nearest water sources.
